# Dual-band transmission polarization converter based on planar-dipole pair frequency selective surface

**DOI:** 10.1038/s41598-018-22092-4

**Published:** 2018-02-28

**Authors:** Shen-Yun Wang, Wei Liu, Wen Geyi

**Affiliations:** Research Center of Applied Electromagnetics, Nanjing University of Information Science and Technology, Nanjing, 210044 China

## Abstract

A novel linear polarization converter operating in C- and X-bands with high polarization conversion ratio is described and demonstrated based on frequency selective surface. The building element is a planar-dipole pair, which is orthogonally printed on a double-layer substrate and vertically connected by a pair of through-via holes functioning as a quasi-two-wire transmission line coupler. A perforated metal shielding layer is sandwiched between the double-layer structure to only support a transverse electric and magnetic (TEM) mode coupling between the top and bottom dipolar components. The front dipole responds to the incident transverse electric (TE) wave, and sends the induced current into the two-wire transmission line to feed the bottom dipole. The bottom dipole is orthogonal or oriented at an arbitrary angle with respect to the top one, and a resultant outgoing transverse magnetic (TM) wave or arbitrary orientation polarized wave can be achieved. In addition, a bidirectional orthogonal polarization converter is realized by using double orthogonally arranged planar-dipole pairs, which are also printed on the same double-layer substrate.

## Introduction

Controlling and manipulating polarization state of electromagnetic wave at will is highly desired as they find applications in imaging and remote sensing in both microwave and optical regimes^1,2^. In satellite communications, polarization converters can be used to countervail the effect of Faraday rotation caused by the ionosphere^3^. Polarization converters have also been utilized in antenna design where polarization diversity is demanded^4,5^, and it is true that the signal polarization must be changed without any rotation or modification of the original antennas for their heavy configuration and complex feed components.

Conventional converter in optical regime, such as wave plate^6,7^, is typically realized by using birefringence effect of some anisotropic materials. Owing to the weak anisotropy of natural materials, the wave plate requires a relatively thick slab compared with its operating wavelength. In order to develop a compact and light-weight device in microwave and millimeter wave regime, it is obvious that conventional converter designs based on anisotropic materials are too bulky and difficult to integrate into today’s miniaturized system.

In past decades, artificial periodic structures, such as frequency selective surfaces (FSSs), metamaterials, and metasurfaces, have opened up a new avenue towards efficient manipulation of electromagnetic (EM) propagation due to its unusual properties, such as the negative refraction^8,9^, invisible cloaking^10,11^, electromagnetically induced transparencies^12–14^, perfect absorbers^15,16^, and electromagnetic energy harvesters^17–20^. The electromagnetic wave polarization converter is widely investigated by using the concept of FSSs sand metasurfaces. Many structures, such as antenna-filter-antenna module^21^, L-shaped antennas^22^, stacked gratings^23–26^, and polarization dependent resonators^27–29^, have been proposed to realize the wave polarization conversion. Comparing with the conventional polarization converter, the artificially structured converter is ultrathin, miniaturized, and easy for integration. A number of high performance FSSs or metasurface-based polarization converters between linear waves^22–31^, circular waves^32^, linear and circular waves^21,33,34^, and linear and vortex waves^35^ have been realized in both transmission and reflection modes. Among various polarization manipulating devices, linear polarization converter working in transmission mode is perhaps the most important one for potential applications, such as in remote metering, where the orientation of a linearly polarized signal cannot be predicted because of the unknown orientation of the source. A linear polarization converter usually rotates the polarization direction of a linearly polarized electromagnetic wave by 90 degree, making it perpendicular to the original one. To the best of our knowledge, the mechanism of most FSSs or metasurface-based polarization converters is often described by the extrinsic chirality in the propagation direction^27,36,37^ or discrepant phase and amplitude modulations of the orthogonal transmission components based on the near-field electromagnetic coupling^28,38,39^. For the unit cell of metasurface, its size is usually electrically small (or in sub-wavelength scale), which calls for high-precision in fabrication and integration. To effectively realize a FSS polarizer, whose typical dimension of the building element is in a quarter wavelength scale, multilayer structures are usually adopted to obtain high frequency selectivity and polarization rotation^40–42^, because it is theoretically true that the maximal conversion efficiency for transmission through a single layer is 50%, while a double layer transmission converter or a single layer with a ground plane can have 100% polarization conversion efficiency^43^. Such limitation has been validated by single-layer meander line polarizers^44^. Alternatively, frequency selectivity and polarization conversion can be simultaneously achieved by using antenna-filter-antenna (AFA) module, which is only a two-layer structure consisting of a receiving antenna, a nonradiating resonant structure, and a transmitting antenna^21,45^. The aperture-coupled-patch FSS polarization converter is a typical polarizer based on AFA module^21^. As mentioned above, although some high-performance polarization converters in transmission mode with high conversion ratio and wideband have been reported, the building elements must be carefully designed and the structural configuration is usually complicated based on the concept of metasurfaces or multilayer FSSs, leading to high-precision requirements in fabrication and integration in a system.

In this paper, we propose an alternate approach to design linear polarization converters by using a simple kind of AFA module with its building element consisting of a planar-dipole pair and quasi-two-wire transmission line coupler. The conversion mechanism is based on the TEM wave coupling using the through-via pair instead of the high-order aperture coupling. Based on the TEM wave coupling with the incident TE wave and outgoing TM wave, dual-band linear polarization converters operating in C- and X-bands with very high conversion efficiency and low insertion loss are achieved.

## Results

### Unit cell design

#### Single linear polarization converters

**Figure 1:**
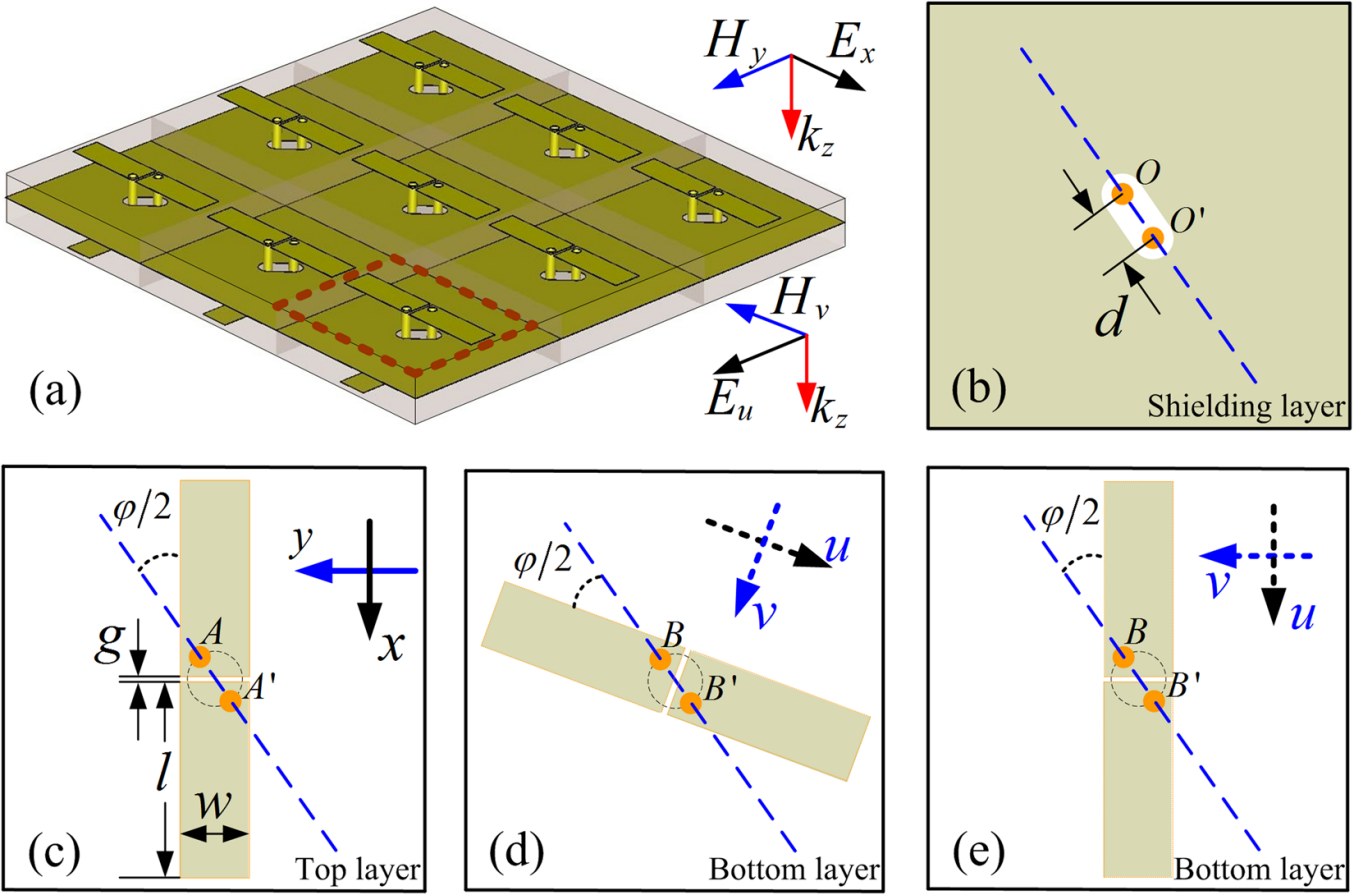
(**a**) Scheme of the sample array, and the top views of the (**b**) shielding layer, (**c**) top layer, (**d**) bottom layer with rotated dipole and (**e**) bottom layer with non-rotated dipole.

Motivated by the aperture-coupled-patch FSS^21^, a class of FSSs by using dipole-coupler-dipole elements is proposed, as shown in Fig. 1(a). The unit cell of conventional aperture-coupled-patch FSS consists of two back-to-back patches mounted on a double-layer substrate, and a common ground plane with a small aperture, such that the two patches are coupled through the small aperture to achieve a high-order band-pass response. Here we replace the small aperture by quasi-two-wire transmission lines using a pair of vertical through-via holes between the top and bottom dipolar components, where the vertical through-via pair passes through a perforated metal shielding layer as shown in Fig. 1(b). For making an impedance matching between the top and bottom planar dipoles, they should be identically designed and fabricated on the top and bottom substrate, respectively, but except for their orientation. Toward this end, consider the incident plane wave to be polarized along *x*-axis in *xyz*-coordinate system, as illustrated in Fig. 1(a), and the transmission wave to be rotated an angle $\phi$. The top dipolar component must be oriented in *x*-axis so as to effectively couple with the incident wave. The diagonal of the through-via holes must be oriented at an angle $\phi /2$ with respect to the incident wave polarization, as shown in Fig. 1(c), while the bottom dipolar component must be oriented at an opposite angle $\phi /2$ with respect to the diagonal of the through-via holes, as shown in Fig. 1(d). For arbitrary angle rotation of the incident polarization, the orientation of the bottom dipolar component is described along *u*-axis in local *uvz*-coordinate system. The dipolar components combined with the through-via holes determine the selectivity of the frequency response, while the orientation of dipolar components determines the polarizations of the incident and transmission waves. To validate the frequency response and polarization being independent, conventional FSS response with co-polarized transmission is achieved for comparison by resuming the orientation of the bottom dipole with that of the top one, as shown in Fig. 1(e).

**Figure 2:**
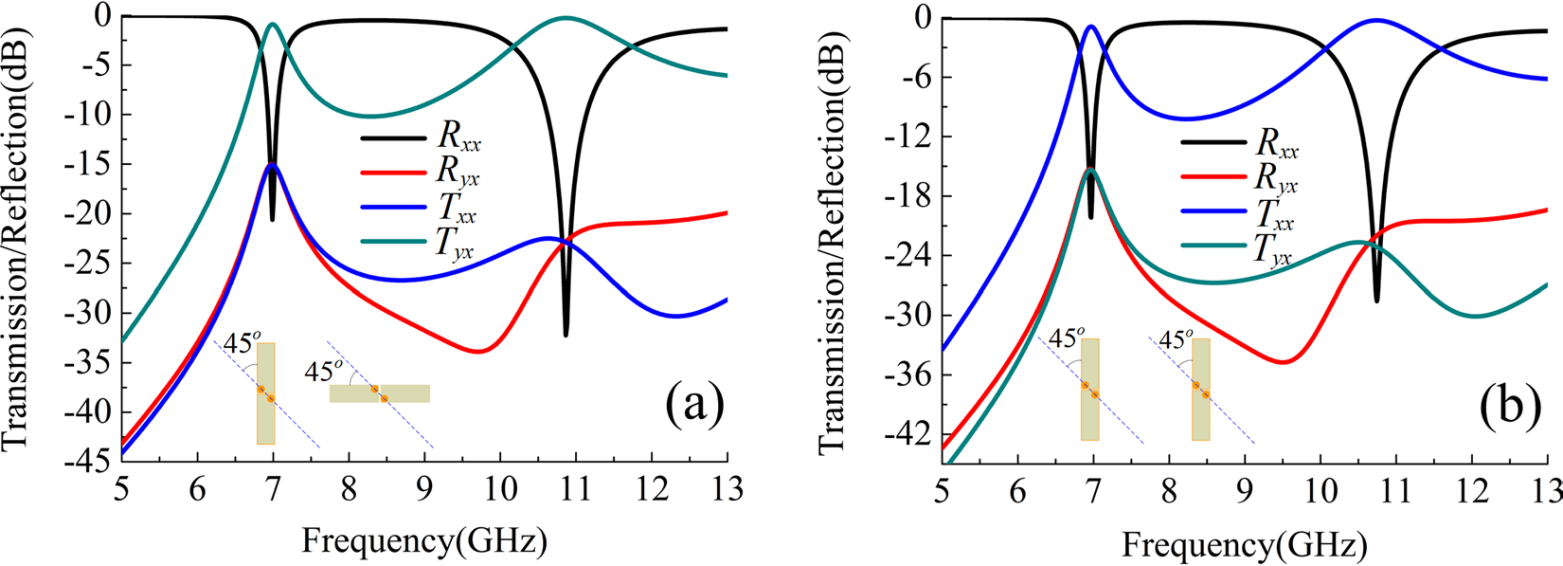
Reflection and transmission for the elements with (**a**) orthogonal planar-dipole pair and (**b**) parallel planar-dipole pair.

At first, we suppose the rotating angle $\phi ={90}^{o}$, a resultant orthogonal polarization converter can be realized. The orthogonal polarization converter is optimized to operate at frequencies of 7.0 and 10.9 GHz, and the dimensions of the element are found to be: *L* = 11.0 mm, *d* = 1.6 mm, *w* = 1.8 mm, *g* = 0.1 mm, *l* = 5.0 mm, *h* = 1.524 mm and *t* = 0.035 mm, where, *L* is the side length of the square unit cell, *h* and *t* represent the thickness of one substrate and metal film, respectively, and the other geometrical parameters are denoted in Figs. 1(b,c). The material of the substrate is Rogers RO4003 with dielectric constant (${\varepsilon }_{d}$) of 3.55 and loss tangent of 0.0027. Such a low-loss double-layer substrate cause low insertion loss for the proposed polarization conversion devices. The optimization of geometrical parameters and electromagnetic simulation are carried out by using commercial software, Ansoft High Frequency Structure Simulator (HFSS), and the frequency domain solver was chosen with periodic boundary condition in the *x*- and *y*-directions and the Floquet ports in the *z*-direction to extract scattering parameters. As the top dipolar component only response to polarized wave in *x*-axis, we define ${R}_{xx}$ and ${R}_{yx}$ as the reflection coefficients of co- and cross-polarized waves, respectively, while ${T}_{xx}$ and ${T}_{yx}$ as the transmission coefficients of co- and cross-polarized waves, respectively (Here, ${R}_{ji}$ or ${T}_{ji}$ denotes *j*-polarized reflection or transmission wave from *i*-polarized incident wave). The amplitudes of co-polarized reflection (${R}_{xx}$) and cross-polarized transmission (${T}_{yx}$) of the proposed orthogonal polarization converter for normal incidence are plotted in Fig. 2(a). It can be seen that a dual pass-band response in cross-polarized transmission mode with working frequency at 7.0 and 10.9 GHz occurs, while the amplitudes of co-polarized transmission (${T}_{xx}$) and cross-polarized reflection (${R}_{yx}$) are both close to zero in the total simulation band. At 7.0 GHz, the cross-polarized transmission coefficient ${T}_{yx}$ is −0.75 dB, and the co-polarized transmission coefficient ${T}_{xx}$ is only −15.2 dB. At 10.9 GHz, the cross-polarized transmission coefficient ${T}_{yx}$ is −0.65 dB, and the co-polarized transmission coefficient ${T}_{xx}$ is −20.2 dB. For comparison, the conventional FSS with dual pass-band response is simulated, where the co-polarized transmission mode occurs at 7.0 GHz and 10.9 GHz, as shown in Fig. 2(b), while the amplitudes of cross-polarized transmission (${T}_{yx}$) and reflection (${R}_{yx}$) are both close to zero in the total simulation band. It is found that the resonant frequency responses are nearly unchanged.

**Figure 3:**
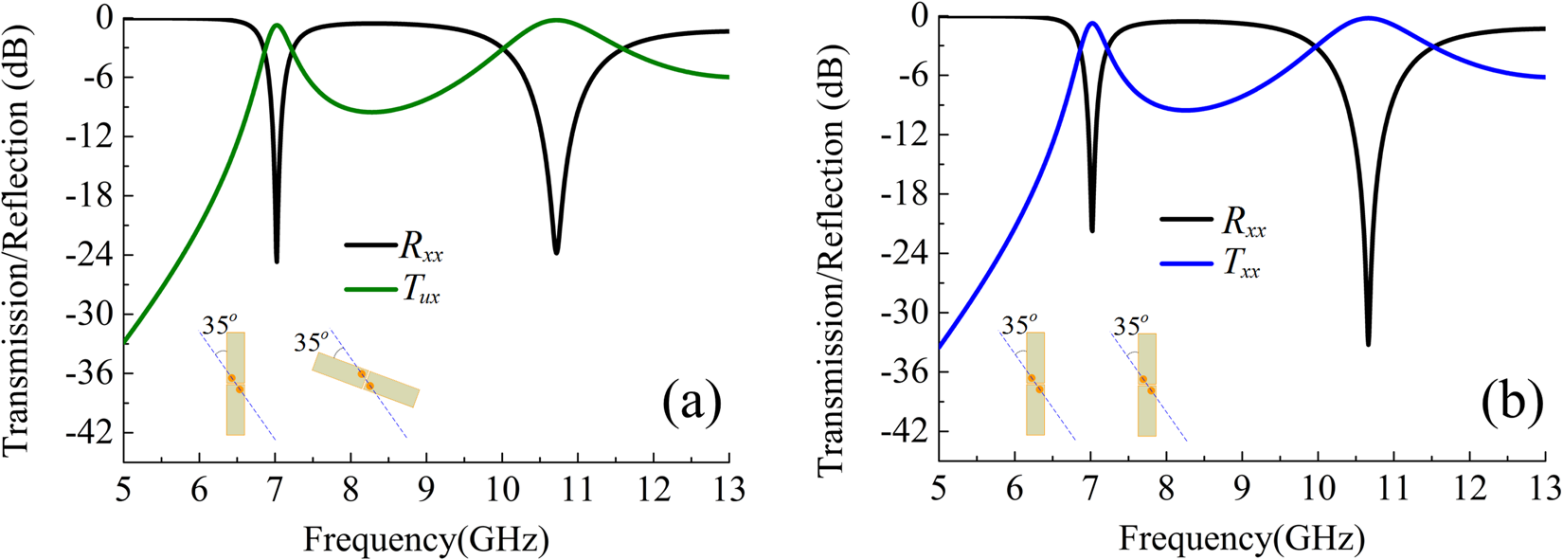
Reflection and transmission for the elements with (**a**) 70-degree-crossed planar-dipole pair and (**b**) parallel planar-dipole pair.

To further validate the design principle, another linear polarization converter with specific rotation angle $\phi ={70}^{o}$ is demonstrated. Keep the all the geometrical parameters unchanged with those of the above example, but the diagonal of the through-via holes must be oriented at an angle $\phi /2={35}^{o}$ with respect to the incident polarization. The orientation of the bottom dipolar component and the polarization of outgoing wave are along *u*-axis in the local *uvz*-coordinate system, thus the polarization converted transmission is represented by ${T}_{ux}$, which is calculated by ${T}_{ux}=\sqrt{{|{T}_{xx}|}^{2}+{|{T}_{yx}|}^{2}}$ in the simulation and shown in Fig. 3(a). One can see that a dual pass-band response is obtained and the resonant frequencies are 7.0 GHz and 10.7 GHz, where the higher resonant frequency is slightly different from that of the orthogonal polarization converter due to difference of azimuth angle of the through-via holes. For comparison, the response of the conventional FSS by assuming the bottom dipole in same orientation with the top one is calculated in Fig. 3(b). Co-polarized transmission mode working at 7.0 GHz and 10.7 GHz can be achieved as well.

#### Bidirectional linear polarization converter

**Figure 4:**
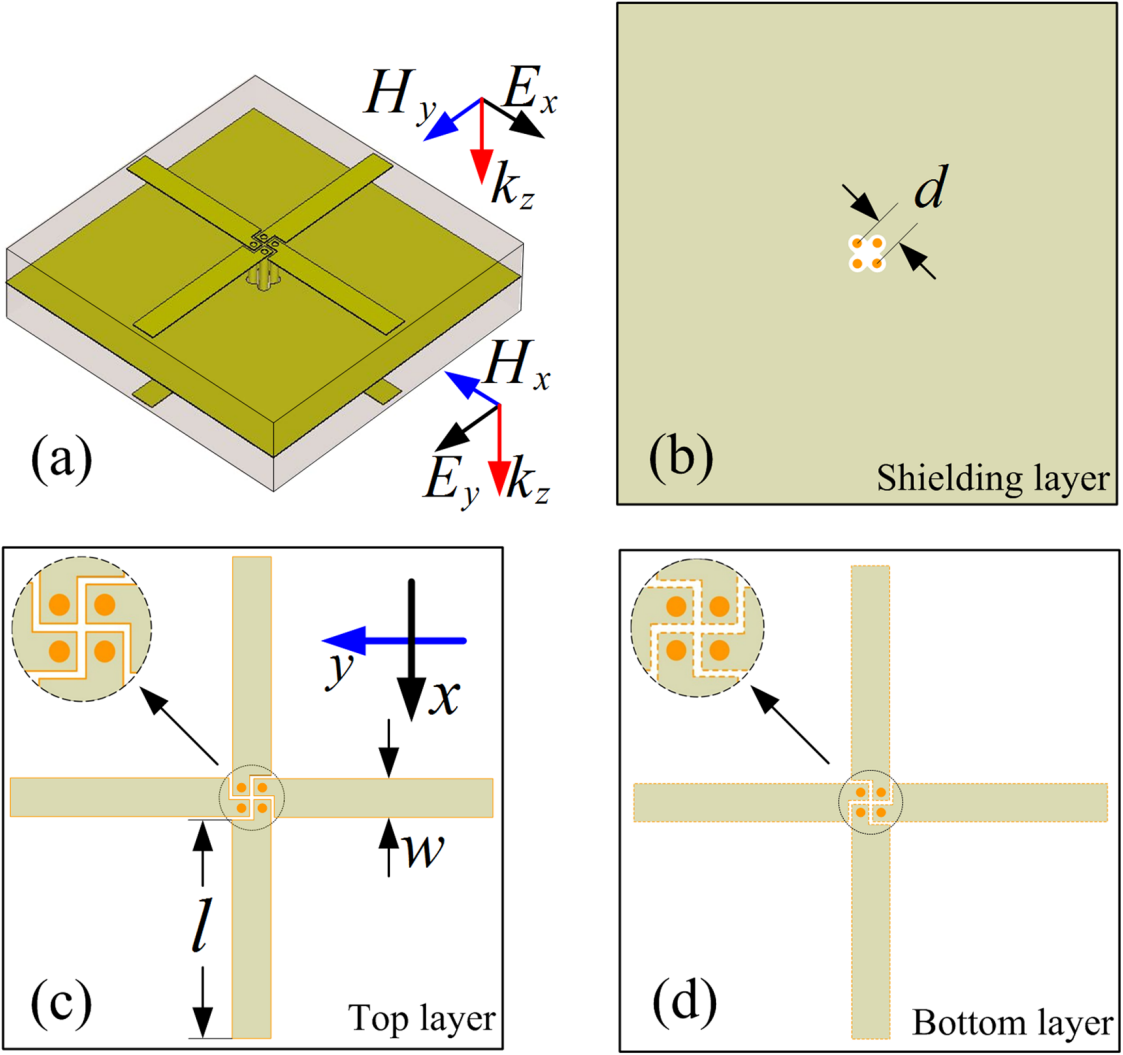
(**a**) Scheme of the sample element, and the top views of the (**b**) shielding layer, (**c**) top layer, (**d**) bottom layer.

**Figure 5:**
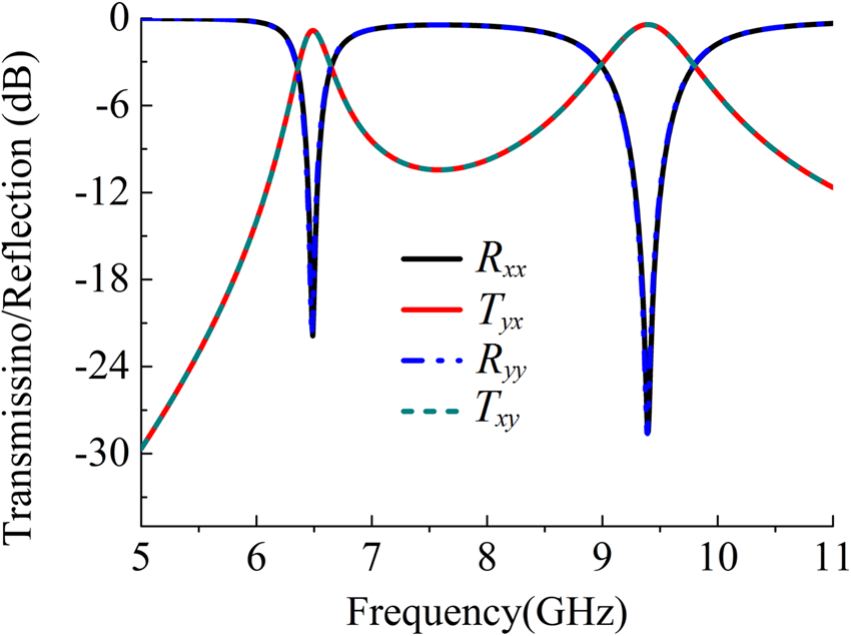
Reflection and transmission of the bidirectional orthogonal polarization converter.

Based on the principle proposed in designing the single linear polarization converter, it is easy to realize the bidirectional linear polarization converters by using double crossed planar-dipole pair elements, as shown in Fig. 4. For simplicity, the element of a bidirectional orthogonal polarization converter is described in Fig. 4(a), which consists of four identical planar dipolar components printed on the same double-layer substrate and connected by four vertical through-via holes. The top views of the shielding layer, top layer and bottom layer are depicted in Figs. 4(b–d), respectively. The width of the dipolar strip is reduced to *w* = 1.0 mm, leading to *l* = 5.0 mm, and *d* = 0.9 mm, as denoted in Figs. 4(b,c). The rest parameters of the building element remain the same as those of the above polarization converters. Finally, we simulated the frequency response when the polarization converter illuminated by incident plane waves with electrical field polarized along *x*-axis and *y*-axis, respectively. Due to the symmetry, the co-polarized reflections in both incident polarizations (${R}_{xx}$ and ${R}_{yy}$) as well as the cross-polarized transmission amplitudes (${T}_{yx}$ and ${T}_{xy}$) are identical, as shown in Fig. 5. We can see that a dual pass-band response appear in cross-polarized transmission mode with working frequency at 6.5 GHz and 9.4 GHz. The resonant frequencies have shifted to lower frequency bands mainly due to the width reduction of the dipolar strip, especially for the higher resonant frequency.

### Experimental results

**Figure 6:**
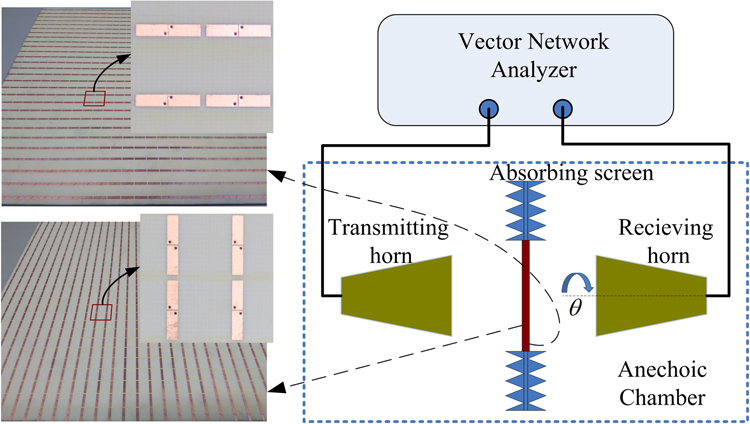
Fabricated device sample and the experimental setup.

One of the prototypes described above, the orthogonal polarization converter, is fabricated and experimentally verified to validate the proposed approach. The conventional printed circuit board technique is utilized and the structural parameters in the fabrication are the same with those of the simulated model. The photograph of the sample is shown in Fig. 6, which has approximate dimensions of 300 × 300 mm, containing 27 × 27 building elements. Measurements are performed in an anechoic chamber, and the fabricated sample is embedded in a microwave absorbing screen to avoid unwanted reflections. The sketch of the experimental setup is also depicted in Fig. 6. The co-polarized reflection ${R}_{xx}$ and cross-polarized transmission ${T}_{yx}$ are measured by using two standard gain ridge-horn antennas connected to a vector network analyzer (N5230A). For setup of the co-polarized reflection measurement, the transmitting and receiving horns are placed adjacently in identical orientation, while the sample is placed in the far-field region of the antennas. The measured reflection coefficient is normalized with respect to that of a metal plane having the same dimensions as the testing sample. During cross-polarized transmission measurement, the antennas are placed in the opposite sides of the sample facing each other in orthogonal polarization. The measured transmission coefficient is also normalized with that of air background. Based on the simulation results, the cross-polarized reflection ${R}_{yx}$ and co-polarized transmission ${T}_{xx}$ are close to zero and their measurements were not carried out.

**Figure 7:**
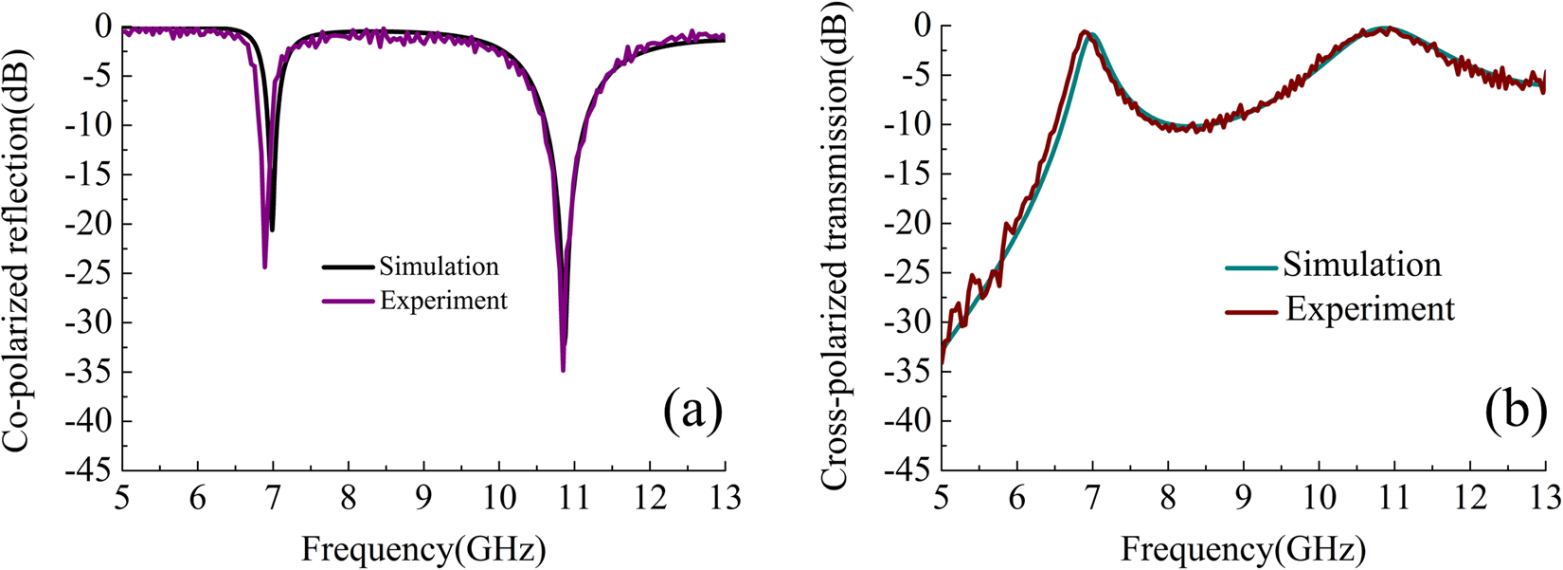
(**a**) Co-polarized reflection and (**b**) cross-polarized transmission obtained from measurement and simulations.

The measured co-polarized reflection and cross-polarized transmission coefficients of the prototype are depicted in Figs. 7(a,b), respectively, and show a good agreement with the simulated responses. The small deviations are likely caused by tolerances of fabrication and measurement.

## Discussion

The principle of the proposed linear polarization converters are discussed based on the prototype of the orthogonal polarization converter. The unit cell is an orthogonally oriented planar dipole pair that is vertically connected by through-via holes. The top planar dipolar component responses to the incident plane wave polarized along *x*-axis (TE wave) and the induced current propagates through the through-via holes to generate a TEM mode at the perforation of the shielding layer. The propagating current is converted into outgoing wave through the bottom dipolar component. The polarization of the outgoing wave is only determined by the orientation of the bottom dipolar component, thus an arbitrary rotation can be realized in principle. To understand the working mechanism, the linear polarization converter is explained by using equivalent circuit model and dipolar antenna theory.

**Figure 8:**
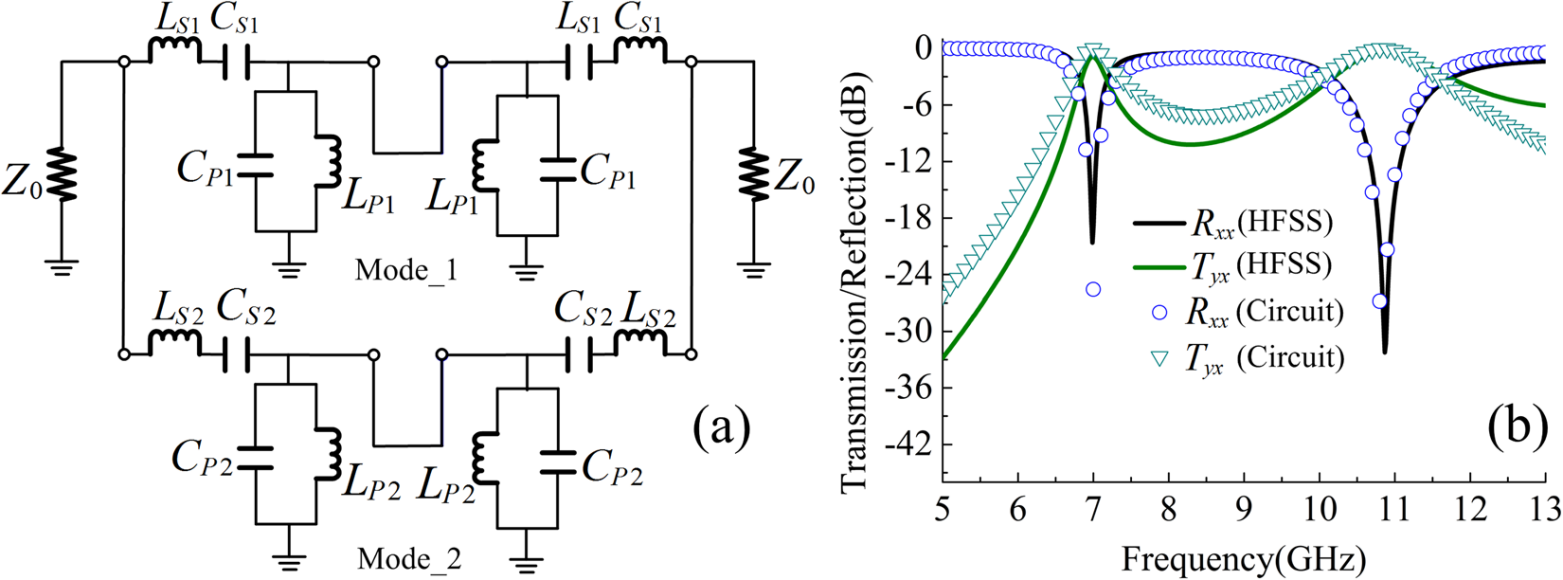
(**a**) The equivalent circuit model of the planar-dipole pair, and (**b**) its reflection and transmission calculated from circuit model and full-wave simulation (HFSS).

The equivalent circuit model of a dipole was developed in refs^46,47^, which consists of a *LC* circuit in series and a *LC* circuit in parallel, where the values of the circuit elements are given to operate at or near their first resonant frequency. Based on this, the proposed linear polarization converter, which consists of a dipole pair and has dual resonant modes, can be represented by the circuit as depicted in Fig. 8(a), where the two parallel circuit branches represent the first and second resonant modes of the linear polarization converter, and the local equivalent circuit for the top and bottom dipolar components are identical within each parallel circuit branch. The values of lumped capacitances and inductances are determined for optimum frequency response. The circuit model simulation in Fig. 8(b) is conducted using the following values: *L*_S1_ = 0.018 nH, *C*_S1_ = 0.635 pF, *L*_P1_ = 12.736 nH, *C*_P1_ = 0.415 pF for the first mode; *L*_S2_ = 0.919 nH, *C*_S2_ = 4.750 pF, *L*_P2_ = 1.332 nH, *C*_P2_ = 0.997 pF for the second mode, and Z_0_ = 377 ${\rm{\Omega }}$ as the characteristic impedance of free space. It is observed that the results from circuit model and HFSS simulations are in good agreement, and the relative errors are acceptable.

**Figure 9:**
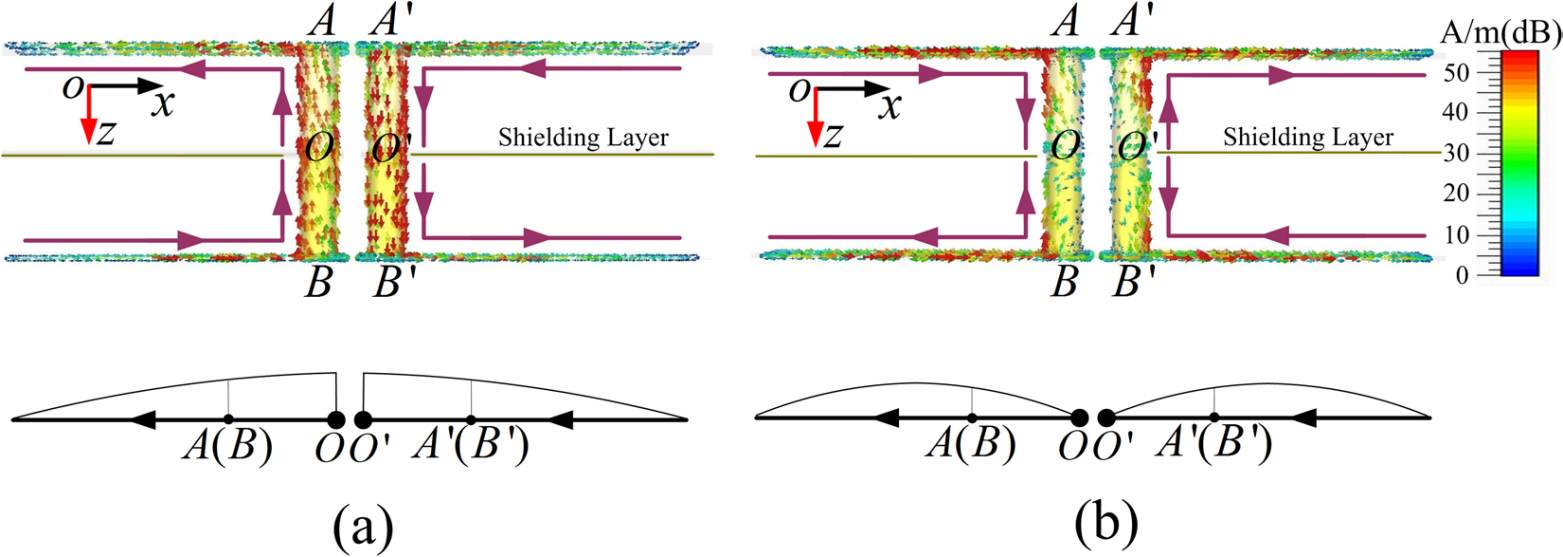
Induced current distribution of the parallel planar-dipole pair for the resonant frequency at (**a**) 7.0 GHz, and (**b**) 10.9 GHz.

**Figure 10:**
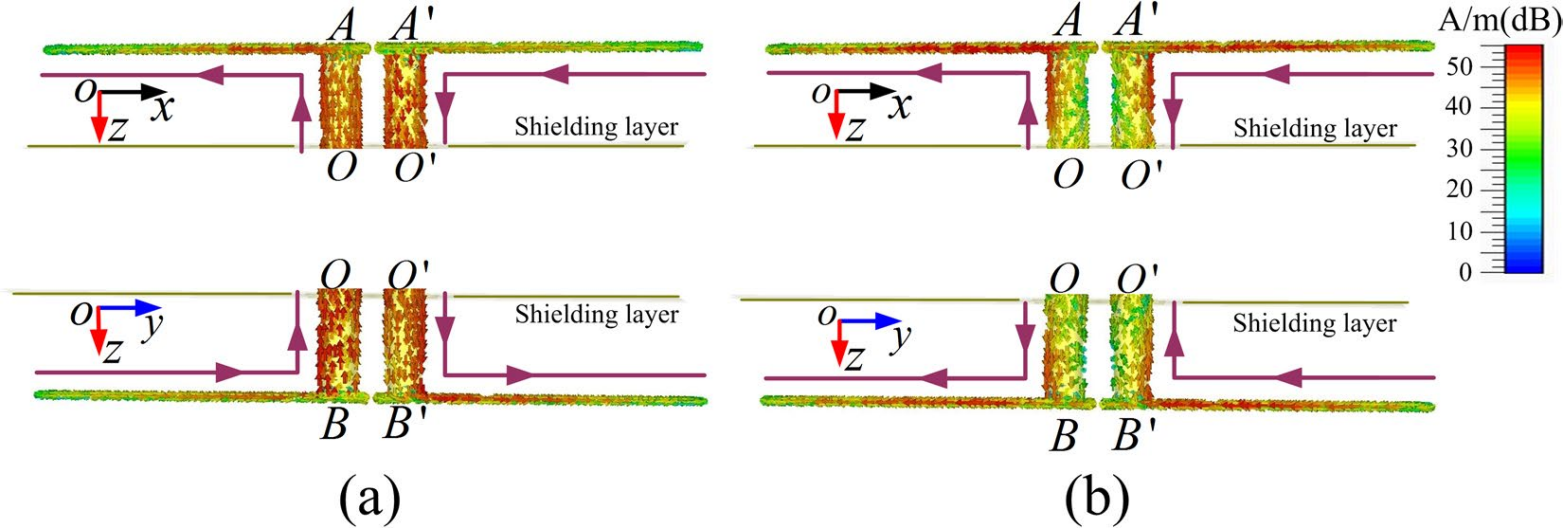
Induced current distribution of the orthogonal planar-dipole pair for the resonant frequency at (**a**) 7.0 GHz, and (**b**) 10.9 GHz.

To further demonstrate the working mechanism of the proposed polarization converter at the two resonant frequency modes, the induced current distributions on the metal components of the conventional FSS response are depicted in Fig. 9, where the top and bottom dipolar components are in same orientation. At 7.0 GHz, both the top and bottom dipolar components function as a half-wavelength dipolar resonator and the maximum induced current occurs at the central part of the through-via holes, as seen in Fig. 9(a). The induced currents on AO (A’O’) and OB (O’B’) are in the same direction. Similarly, at 10.9 GHz, both the top and bottom dipoles can be treated as full wavelength dipolar resonators and the minimum induced current appears at the central part of the through-via holes, as depicted in Fig. 9(b) and the induced currents on AO (A’O’) and OB (O’B’) are in opposite direction. The equivalent dipole models for both the half-wavelength and full-wavelength dipoles are shown in Figs. 9(a,b), respectively. The resonant modes are determined by the total wave paths^48^: $l\cdot \sqrt{{\varepsilon }_{eff}}+h\cdot \sqrt{{\varepsilon }_{d}}=c/2{f}_{01}$ approximately determines the half-wavelength resonance, and $[l+w/2]\sqrt{{\varepsilon }_{eff}}+h\cdot \sqrt{{\varepsilon }_{d}}=c/{f}_{02}$ approximately determines the full wavelength resonance with $c$ being the speed of light in vacuum, ${\varepsilon }_{eff}$ the effective permittivity of the dielectric for the strip lines, ${\varepsilon }_{d}$ the permittivity of the substrate, and the influence of the strip width *w* affects the higher resonant frequency, as mentioned in the proceeding. For the orthogonal polarization converter, the induced current distributions on the metal components for both modes are similar to those depicted in Fig. 9, as shown in Fig. 10. However, the current distributions on the bottom dipole are in the orthogonal plane to realize the cross-polarized transmission as depicted in the bottom parts in Figs. 10(a,b). The rotation of the bottom dipole component causes no change of the frequency response, so that the crossed dipole pair can also be treated as half-wavelength and full-wavelength dipolar resonators at 7.0 GHz and 10.9 GHz, respectively. The dual resonant current distributions for polarization converters with arbitrary rotation angle and bidirectional orthogonal conversions are similar, and are not discussed here.

The concept of AFA module proposed in this paper is similar to antenna–delay line–antenna elements to form discrete transmitting arrays^49,50^. The demonstrated linear polarization converter based on dipolar-pair FSS is useful in sensing and imaging systems. For instance, two orthogonal polarization waves can be obtained from a linear polarized microwave source, and a clearer reflection image can be reconstructed by combining the individual transmission images of the polarization sensitive objects.

## Method

### Numerical simulation

The electromagnetic simulation is carried out by the frequency domain solver of commercially available software, Ansoft High Frequency Structure Simulator (HFSS). In the simulations, the unit cell boundary conditions are used along *x-* and *y-*directions, and the absorbing boundary condition is applied in the *z*-direction. The model is excited by Floqued port with the normal incidence of linearly polarized waves in the frequency range of 5.0GHz-13.0 GHz.

### Sample fabrication

The sample is built on a multilayered printed circuit board. Both the top and bottom copper layers with a thickness of 0.035 mm are printed on two Rogers RO4003 layers with a thickness of 1.524 mm. A perforated copper film is then sandwiched between the two Rogers RO4003 layers using adhesive (whose dielectric constants are almost the same as Rogers RO4003) with a thickness of 0.035 mm to suppress the near-field coupling between the top and bottom unit resonators. Finally, the through-via holes are fabricated by using standard coppering and drilling methods.

## References

[1] Mertens K, Scholl B, Schmitt HJ (1998). Strong polarization conversion in periodically loaded strip waveguides. IEEE Photonics Techl..

[2] Dietlein C, Luukanen A, Popovi Z, Grossman E (2007). A w-band polarization converter and isolator. IEEE Trans.Antennas Propag..

[3] Maral G. & Bousquet, M. Satellite Communications Systems: Systems, Techniques and Technology (ed. Sun, Z.) Ch. 5, 208 *(Wile*y, 2009).

[4] Ma X (2014). A dual circularly polarized horn antenna in Ku-band based on chiral metamaterial. IEEE Trans. Antennas Propag..

[5] Ma X (2012). Single-layer circular polarizer using metamaterial and its application in antenna. Microw. Opt. Techn. Lett..

[6] Masson JB, Gallot G (2006). Terahertz achromatic quarter-wave plate. Opt. Lett..

[7] Chen CY, Tsai TR, Pan CL, Pan RP (2003). Room temperature terahertz phase shifter based on magnetically controlled birefringence in liquid crystals. Appl. Phys. Lett..

[8] Smith DR, Padilla WJ, Vier DC, Nemat-Nasser SC, Schultz S (2000). Composite medium with simultaneously negative permeability and permittivity. Phys. Rev. Lett..

[9] Shelby RA, Smith DR, Schultz S (2001). Experimental verification of a negative index of refraction. Science.

[10] Schurig D (2006). Metamaterial electromagnetic cloak at microwave frequencies. Science.

[11] Liu R (2009). Broadband ground-plane cloak. Science.

[12] Li HM (2015). Electromagnetically induced transparency with large delay-bandwidth product induced by magnetic resonance near field coupling to electric resonance. Appl. Phys. Lett..

[13] Li HM (2015). Low-loss metamaterial electromagnetically induced transparency based on electric toroidal dipolar response. Appl. Phys. Lett..

[14] Li HM (2016). Tailoring electromagnetically induced transparency with different coupling mechanisms. Sci. Rep..

[15] Landy NI, Sajuyigbe S, Mock JJ, Smith DR, Padilla WJ (2008). Perfect metamaterial absorber. Phys. Rev. Lett..

[16] Bian B, Liu S, Wang S, Kong X (2013). Novel triple-band polarization-insensitive wide-angle ultra-thin microwave metamaterial absorber. J. Appl. Phys..

[17] Xie Y (2014). A universal electromagnetic energy conversion adapter based on a metamaterial absorber. Sci. Rep..

[18] Ramahi OM, Almoneef TS, Alshareef M, Boybay MS (2012). Metamaterial particles for electromagnetic energy harvesting. Appl. Phys. Lett..

[19] Xu P, Wang SY, Geyi W (2016). Design of an effective energy receiving adapter for microwave wireless power transmission application. AIP Advances.

[20] Wang SY, Xu P, Geyi W, Ma Z (2016). Split-loop resonator array for microwave energy harvesting. Appl. Phys. Lett..

[21] Lin B, Wu JL, Da XY, Li W, Ma JJ (2017). A linear-to-circular polarization converter based on a secondorder band-pass frequency selective surface. Appl. Phys. A.

[22] Lévesque Q (2014). Plasmonic planar antenna for wideband and efficient linear polarization conversion. Appl. Phys. Lett..

[23] Grady NK (2013). Terahertz metamaterials for linear polarization conversion and anomalous refraction. Science.

[24] Liu DJ, Xiao ZY, Ma XL, Wang ZH (2015). Broadband asymmetric transmission and multi-band 90° polarization rotator of linearly polarized wave based on multi-layered metamaterial. Opt. Commun..

[25] Zhou G (2016). Designing perfect linear polarization converters using perfect electric and magnetic conducting surfaces. Sci. Rep..

[26] Chen H (2016). Ultra-wideband transparent 90° polarization conversion metasurfaces. Appl. Phys. A.

[27] Chen K (2017). Dynamic control of asymmetric electromagnetic wave transmission by active chiral metamaterial. Sci. Rep..

[28] Shi H, Zhang A, Zheng S, Li J, Jiang Y (2014). Dual-band polarization angle independent 90° polarization rotator using twisted electric-field-coupled resonators. Appl. Phys. Lett..

[29] Chin JY, Lu M, Cui TJ (2008). Metamaterial polarizers by electric-field-coupled resonators. Appl. Phys. Lett..

[30] Ling F, Yao G, Yao J (2016). Active tunable plasmonically induced polarization conversion in the THz regime. Sci. Rep..

[31] Woo JM, Hussain S, Jang JH (2017). A terahertz in-line polarization converter based on through-via connected double layer slot structures. Sci. Rep..

[32] Zalkovskij M (2013). Optically active Babinet planar metamaterial film for terahertz polarization manipulation. Laser Photonics Rev..

[33] Ma X (2014). An active metamaterial for polarization manipulating. Adv. Opt. Mater..

[34] Li Z, Liu W, Hua C, Chen S, Tian J (2015). Realizing broadband and invertible linear-to-circular polarization converter with ultrathin single-layer metasurface. Sci. Rep..

[35] Yang Y (2014). Dielectric meta-reflectarray for broadband linear polarization conversion and optical vortex generation. Nano Lett..

[36] Rajkumar R, Yogesh N, Subramanian V (2013). Cross polarization converter formed by rotated-arm-square chiral metamaterial. J. Appl. Phys..

[37] Zhu W, Rukhlenko ID, Xiao F, Premaratne M (2014). Polarization conversion in u-shaped chiral metamaterial with four-fold symmetry breaking. J. Appl. Phys..

[38] Tremain B, Rance HJ, Hibbins AP, Sambles JR (2015). Polarization conversion from a thin cavity array in the microwave regime. Sci. Rep..

[39] Zhang L (2016). Ultrabroadband design for linear polarization conversion and asymmetric transmission crossing x- and k-band. Sci. Rep..

[40] Li L, Li Y, Wu Z, Zhang Y, Zhao G (2015). Novel Polarization reconfigurable converter based on multilayer frequency-selective surfaces. IEEE Proc..

[41] Abadi SMAMH, Behdad N (2016). Wideband linear-to-circular polarization converters based on miniaturized-element frequency selective surfaces. IEEE Trans. Antennas Propag..

[42] Abadi SMAMH, Behdad N (2016). A broadband, circular-polarization selective surface. J. Appl. Phys..

[43] Markovich DL, Andryieuski A, Zalkovskij M, Malureanu R, Lavrinenko AV (2013). Metamaterial polarization converter analysis: limits of performance. Appl. Phys. B.

[44] Fei P, Shen Z, Wen X, Nian F (2015). A single-layer circular polarizer based on hybrid meander line and loop configuration. IEEE Trans. Antennas Propag..

[45] Abbaspour-Tamijani A, Sarabandi K, Rebeiz M (2004). Antenna-filter-antenna arrays as a class of bandpass frequency-selective surfaces. IEEE Trans. Antennas Propag..

[46] Hamid M, Hamid R (1997). Equivalent circuit of dipole antenna of arbitrary length. IEEE Trans. Antennas Propag..

[47] Liao Y, Hubing TH, Su D (2012). Equivalent circuit for dipole antennas in a lossy medium. IEEE Trans. Antennas Propag..

[48] Winkler SA, Hong W, Bozzi M, Wu K (2010). Polarization rotating frequency selective surface based on substrate integrated waveguide technology. IEEE Trans. Antennas Propag..

[49] Kaouach H, Dussopt L, Lantéri J, Koleck T, Sauleau R (2011). Wideband low-loss linear and circular polarization transmit-arrays in V-band. IEEE Trans. Antennas Propag..

[50] Clemente A, Dussopt L, Sauleau R, Potier P, Pouliguen P (2013). Wideband 400-element electronically reconfigurable transmitarray in Xband. IEEE Trans. Antennas Propag..

